# Actiflagelin, a new sperm activator isolated from *Walterinnesia aegyptia* venom using phenotypic screening

**DOI:** 10.1186/s40409-018-0140-4

**Published:** 2018-01-23

**Authors:** Tarek Mohamed Abd El-Aziz, Sawsan Al Khoury, Lucie Jaquillard, Mathilde Triquigneaux, Guillaume Martinez, Sandrine Bourgoin-Voillard, Michel Sève, Christophe Arnoult, Rémy Beroud, Michel De Waard

**Affiliations:** 1 Institute of Thorax, INSERM UMR 1087/CNRS UMR 6291, LabEx Ion Channels, Science and Therapeutics, 8 Quai Moncousu, BP 70721, 44007 Nantes Cedex 1, France; 2 University of Nantes, 44007 Nantes, France; 3 Zoology Department, Faculty of Science, Minia University, El-Minia, 61519 Egypt; 4 Smartox Biotechnology, 570 Rue de la Chimie, 38400 Saint Martin d’Hères, France; 5 University Grenoble Alpes, PROMETHEE proteomic Platform, 38000 Grenoble, France; 6 INSERM 1209, CNRS UMR 5309, Equipe “Génétique, Epigénétique et Thérapies de l’Infertilité”, 38000 Grenoble, France; 7 Institut de Biologie et de Pathologie, CHU de Grenoble, PROMETHEE proteomic Platform, 38000 Grenoble, France; 8 Inserm U1055, LBFA and BEeSy, Saint Martin d’Hères, France

**Keywords:** Snake venom, *Walterinnesia aegyptia*, Bioactive compounds, Fertility, Sperm motility, Venomics, Tandem mass spectrometry, De novo sequencing, Edman degradation

## Abstract

**Background:**

Sperm contains a wealth of cell surface receptors and ion channels that are required for most of its basic functions such as motility and acrosome reaction. Conversely, animal venoms are enriched in bioactive compounds that primarily target those ion channels and cell surface receptors. We hypothesized, therefore, that animal venoms should be rich enough in sperm-modulating compounds for a drug discovery program. Our objective was to demonstrate this fact by using a sperm-based phenotypic screening to identify positive modulators from the venom of *Walterinnesia aegyptia*.

**Methods:**

Herein, as proof of concept that venoms contain interesting compounds for sperm physiology, we fractionated *Walterinnesia aegyptia* snake venom by RP-HPLC and screened for bioactive fractions capable of accelerating mouse sperm motility (primary screening). Next, we purified each compound from the positive fraction by cation exchange and identified the bioactive peptide by secondary screening. The peptide sequence was established by Edman sequencing of the reduced/alkylated compound combined to LC-ESI-QTOF MS/MS analyses of reduced/alkylated fragment peptides following trypsin or V8 protease digestion.

**Results:**

Using this two-step purification protocol combined to cell phenotypic screening, we identified a new toxin of 7329.38 Da (actiflagelin) that activates sperm motility in vitro from OF1 male mice. Actiflagelin is 63 amino acids in length and contains five disulfide bridges along the proposed pattern of disulfide connectivity C_1_-C_5_, C_2_-C_3_, C_4_-C_6_, C_7_-C_8_ and C_9_-C_10_. Modeling of its structure suggests that it belongs to the family of three finger toxins with a noticeable homology with bucandin, a peptide from *Bungarus candidus* venom.

**Conclusions:**

This report demonstrates the feasibility of identifying profertility compounds that may be of therapeutic potential for infertility cases where motility is an issue.

## Background

Mammalian fertilization is the result of the successful union of two gametes. Global reports indicate that infertility concerns 15% of all couples worldwide [1]. Infertility is, therefore, becoming a growing concern in modern society. It is estimated that 30% of all infertility cases are due to male factors, namely reduced sperm count, decreased motility (asthenozoospermia) and abnormal morphology. Complete asthenozoospermia (cases of no motility at all) are reported at a frequency of 1 in 5000 men [2, 3].

The molecular pathways and sperm proteins involved in the three main physiological functions of mature sperm that occur in the female tract – sperm motility, capacitation and acrosome reaction – are still the subject of intensive research activities [4, 5]. In spite of possessing a complete understanding of the cascade of events that preside over these sperm functions, it appears clear that subtle interplays between cell surface receptors and ion channels play essential contributing roles to these processes [6–8]. This holds particularly true for sperm motility, which involves a coordinated movement of the flagellum [9–11]. As G-protein coupled receptors (GPCR) and ion channels alone account for the first and second most important targets, as witnessed by currently marketed drugs [12], it would come as no surprise that screening programs with libraries enriched in compounds active on these pharmacological targets should impact these sperm functions.

Herein, we took advantage of our knowledge on animal venoms to develop the proof of concept that bioactive natural peptides may facilitate sperm motility. Ultimately, we hope that some of these compounds have the potential to treat asthenozoospermia. Earlier efforts in using animal venoms to discover compounds that interfere with sperm functions had led to several interesting discoveries. For instance, it was found that snake venoms are rich sources of secreted phospholipase A_2_ that influences sperm motility, acrosome reaction and in vitro fertilization [13]. This was the case for *Oxyuranus scutellatus scutellatus* snake venom*.* Using this lead approach, it also unusually shed light on the importance of mammalian group X phospholipase A_2_, which is secreted by sperm itself during the acrosome reaction, in positively favoring the outcome of fertilization while concomitantly decreasing sperm motility [14, 15]. More recently, using a venom-targeted screening program, Martinez and collaborators [16] identified spermaurin from *Maurus palmatus* scorpion venom as a compound improving sperm motility and fertilization in various species. Therefore, there is no doubt that animal venoms should contain many more compounds of interest to modulate spermatic functions in vitro and in vivo.

Among other interesting features of animal venoms as source of compounds, it is worth mentioning the fact that they are intrinsically tailored for in vivo use with very little or no degradation observed upon in vivo administration [17]. Therefore, it is not surprising that many blockbuster therapeutic agents are derived from peptides originally identified in animal venoms. Most evident examples include: captopril, a FDA approved inhibitor of angiotensin-converting enzyme, for hypertension [18]; and eptifibatide, an antiplatelet agent isolated from *Sistrurus miliarius barbouri* venom, which inhibits fibrinogen from binding to platelet glycoprotein IIb/IIIa receptor [19].

With the advent of venomics, a set of proteomic analyses aiming at identifying the compounds present in venoms, drug discovery can be significantly accelerated. These technologies are best backed up with efficient separation techniques such as reversed-phase high performance liquid chromatography (RP-HPLC) – that separates the bioactive compounds based on their hydrophobicity – and ion exchange chromatography – that fractionates them according to their net charge. A two-step separation procedure is generally sufficient to isolate compounds of interest and determine their mass by matrix-assisted laser desorption/ionization-time of flight mass spectrometry (MALDI-TOF MS) or liquid chromatography electrospray ionization quadrupole time-of-flight mass spectrometry (LC-ESI-QTOF MS) [20] and sequence identity by MS/MS techniques. Besides, Edman degradation remains a method of choice to identify the amino acid sequence of a peptide [21]. During one sequencing run, more than 30 amino acids can be determined in average, although in many cases the N-terminal may be modified or blocked preventing Edman degradation to work. This is the case for 1.5% of spider toxins that possess a N-terminus sequence blocked with pyroglutamate [22].

For the current project, we postulated that we should be able to identify de novo a peptide sequence from a single snake venom active on sperm motility. We reasoned that since so many receptors and channels control sperm motility and that venoms are rich in compounds active on these targets, we should be able to identify a compound without even aiming at diversifying the sources of animal venoms. For proof of concept, we used *Walterinnesia aegyptia* black desert cobra venom*,* a venomous Elapidae snake from desert areas of Africa and Middle East. While its venom is known to cause muscular paralysis and respiratory difficulties in mice, only two neurotoxins have been identified so far: a phospholipase A_2_ and an acetylcholinesterase [23–26]. While therapeutic potential of this venom has been illustrated by its antitumor efficacy in vitro on human breast carcinoma cell lines [27], it has never been used on human populations to treat infertility issues. For this study on sperm motility, we benefited from the experience built up on the best fractionation techniques to separate the compounds of this venom [28]. Herein, we report on the identification of actiflagelin, a new sperm motility activator using advanced techniques in venomics.

## Methods

### Materials

The lyophilized venom of a single Egyptian black desert snake *W. aegyptia* (Elapidae) was purchased from Alphabiotoxine Laboratory (Montroeul-au-bois, Belgium). The snake originates from the Sinai Peninsula region of Egypt. Sperm motility M2 medium was purchased from Sigma Aldrich (Saint-Quentin Fallavier, France). Standard count chamber slides (two chambers slides, 100 μm depth) were from Leja Products (Netherlands). The proteolytic enzymes were from Sigma Aldrich (trypsin) and Promega (V8 protease).

### RP-HPLC fractionation of the snake venom

The crude venom was fractionated using an analytical RP-HPLC C18 column (XBridge™ BEH 130, 3.5 μm and 4.6 mm ID × 250 mm L column) attached to an Agilent 1260 HPLC (Agilent Technologies). Two mobile phases – A [0.1 trifluoroacetic acid (TFA)/99.9 distilled H_2_O (dH_2_O) (*v*/v)] and B [0.1 TFA/90 acetonitrile (ACN)/9.9 dH_2_O (v/v/v)] – were used for the RP-HPLC fractionation. Lyophilized snake venom (18 mg) was dissolved in 300 μL of buffer A and filtered before injection into the RP-HPLC system. Elution of venom components was performed at a flow rate of 0.5-2 mL/min with a 5-80% gradient of B/A buffers. Several fractions were collected automatically, lyophilized and stored at −20 °C. For mouse sperm motility assay, RP-HPLC fractions were resuspended in 100 μL distilled water.

### Cation exchange chromatography and desalting of the cation exchange major peaks

The sperm motility assay-positive RP-HPLC fraction was further separated by cation exchange chromatography. The lyophilized RP-HPLC fraction was dissolved in buffer A (20 mM of CH_3_COONa, pH 4.5) and injected to a TOSOH Bioscience column (TSK gel SP-STAT, 7 μm, 4.6 mm ID × 10 cm L, TOSOH Bioscience, Germany) coupled to an Agilent 1260 HPLC (Agilent technologies). The elution of fraction components was performed with a linear salt gradient from 0 to 1 M of buffers C/A (buffer C composition: 20 mM CH_3_COONa, 1 M NaCl, pH 4.5) over 30 min. Major peaks were collected automatically and were purified using RP-HPLC. Desalting of the cation exchange chromatography major peaks were performed on RP-HPLC C18 advanced Bio-peptide column (250 mm × 2.1 mm, 2.7 μm) coupled to Agilent 1260 HPLC. The desalted peak was collected automatically, lyophilized and stored at −20 °C. For mouse sperm motility assays, the desalted peaks were resuspended in 100 μL distilled water.

### Computer assisted semen analysis (CASA)

All the fractions were tested on mouse sperm solution using computer-assisted semen analysis (CASA) to examine the sperm motility parameters. The two caudae epididymis of 2-4 months-old OF1 male mice (Charles River, France) were isolated by dissection. Spermatozoa from the epididymis were allowed to swim in 1 mL of M2 medium for 10 min at 37 °C. Sperm solution were diluted in M2 medium (ratio 1:50; Sigma-Aldrich) and incubated with RP-HPLC fractions or cation exchange major peak fractions at 37 °C for 10 min (190 μL of sperm solution were mixed with 10 μL of each fraction). After incubation, 25-μL sperm solution was immediately transferred into a standard count chamber slide and kept at 37 °C for microscopic measurement of sperm movement.

Sperm motility parameters were measured using a sperm analyzer (Hamilton Thorn Research, Beverley). The settings employed for analysis were as follows: acquisition rate, 60 Hz; number of frames, 60; minimum contrast, 30; minimum cell size, 4; cell intensity, 75; path velocity, 50; and magnification factor, 0.70. The motility parameters measured were the curvilinear velocity (VCL) and the amplitude of the lateral head displacement (ALH). In addition, we also measured the average path velocity (VAP) and the straight-line velocity (VSL). A minimum of 40 motile spermatozoa were analyzed for each assay. Sperms were their own controls, which explains why two mice were sufficient for this investigation. The comparison between groups of sperms arising from different animals was far less reliable than the protocol described herein.

### Amino acid sequence characterization

#### Sample preparation

All of the screening-positive purified venom peptide obtained after the two chromatography steps was resuspended in 100 mM ammonium bicarbonate (pH 8), reduced with Tris (2-carboxyethyl) phosphine hydrochloride (TCEP, incubated at 55 °C for 1 h) and alkylated with iodoacetamide (incubated at room temperature in dark for 1 h) prior to enzyme digestion. The reduced/alkylated venom peptide was digested by using one of the following complementary enzymes: trypsin and V8 protease. The enzyme was added at a 1:20 ratio (enzyme/peptide, *w*/w) and incubated overnight at 37 °C. The enzymatic digestion was stopped by 0.1% formic acid.

#### LC-ESI-QTOF MS/MS analysis

A Waters Q-TOF Xevo G2S mass spectrometer equipped with an Acquity UHPLC system and Lockspray source was used for the acquisition of the LC-ESI-QTOF MS and MS/MS data. The digested peptide sample was injected into an Acquity UPLC BEH300 C18 column (1.7 μm, 2.1 mm ID × 150 mm L, Waters). The elution of the peptides was performed with a 10-70% gradient of buffers B/A over 10 min [solvent A composition: dH_2_O/formic acid, 99.9/0.1 (*v*/v) and solvent B composition: ACN/formic acid, 99.9/0.1 (v/v)]. The eluted solution was directly injected into the coupled MS system. Acquisition and analysis of the peptide samples were carried out in the positive mode, within a mass range of *m/z* 100-2000 using the Agilent MassLynx software version 4.1 (Waters).

The mass spectrometer settings for the MS analyses were: capillary voltage, 0.5 kV; cone voltage, 40 V; source temperature, 150 °C; desolvation temperature, 600 °C; gas flow, 80 L/h; and gas desolvation, 1000 L/h. MS data were acquired using a data-dependent acquisition method (DDA) for which MS/MS data were acquired using CID activation mode based on mass and charge state of the candidate ions. For calibration, an external lock mass was used with a separate reference spray (LockSpray) using a solution of leucine enkephalin eluted at a flow rate of 5 μL/min. The calibration was based on the MS detection of *m/z* 278.1141 and 556.2771 ions at collision energy of 23 eV. The resulting MS/MS spectra data were analyzed by de novo sequencing using PEAKS® studio version 5.2 software (Bioinformatics Solutions Inc.) with the following settings: trypsin, or V8 protease; carbamidomethyl (C) as a fixed modification; mass accuracy for MS/MS data at 0.05 Da; and mass accuracy for the precursor mass at 10 ppm. Amino acid sequence scores between 50 and 100 were recorded.

#### MALDI-TOF MS analysis

The venom fraction 11 was analyzed by MALDI-TOF MS using the 4800 MALDI TOF/TOF Analyzer mass spectrometer (Sciex, France). The 0.5 μL volume sample of the fraction was spotted on the MALDI plate and mixed with 0.5 μL MALDI matrix (CHCA at 10 mg/mL in 70.0% ACN, 29.9% distilled H_2_O, and 0.1% TFA). The MS spectrum was recorded in positive linear middle mass mode (*m/z* 2000-20,000, mass tolerance 5 *m/z* units).

#### N-terminal sequence determination

Amino acid sequence determination based on Edman degradation was performed using an Applied Biosystems gas-phase sequencer model 492 (s/n: 9510287J). Phenylthiohydantoin amino acid derivatives generated at each sequence cycle were identified and quantitated on-line with an Applied Biosystems Model 140C HPLC system using the data analysis system for protein sequencing from Applied Biosystems (software Procise PC v2.1). The PTH-amino acid standard kit (Perkin-Elmer P/N 4340968) was used and reconstituted according to the manufacturer’s instructions. The procedures and reagents used were as recommended by the manufacturer. Chromatography was used to identify and quantify the derivatized amino acid removed at each sequence cycle. Retention times and integration values of peaks were compared to the chromatographic profile obtained for a standard mixture of derivatized amino acids.

### Search of peptides analogues

The highest scoring sequences of the identified peptides were compared to the protein databases (SwissProt, BLASTP 2.6.1+) through the BLAST website (http://blast.ncbi.nlm.nih.gov) for protein similarities. Only proteins from the database with high identity percentages (at least 60%) were selected. The theoretical molecular masses of the identified peptides were calculated online using ExPASy ProtParam website (https://web.expasy.org/protparam).

### Structural modelling of actiflagelin

The 3D structure of actiflagelin was modeled using the SwissModel automatic modeling protein software (http://swissmodel.expasy.org).

## Results

**Fig. 1:**
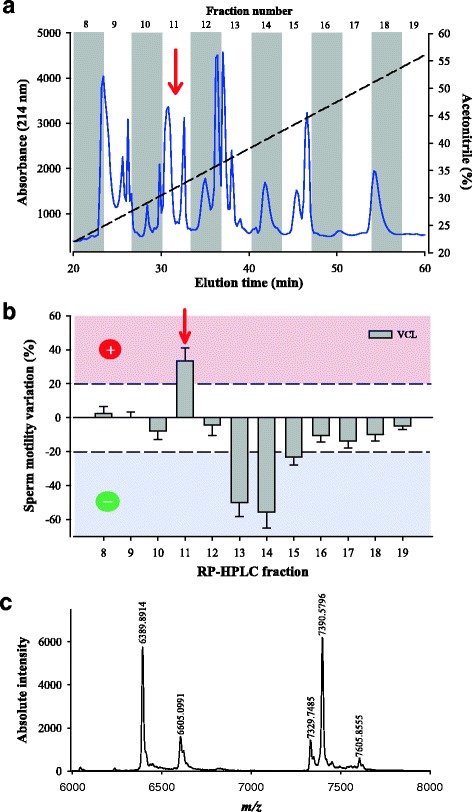
Bioactivity of *W. aegyptia* venom primary fractions on OF1 mouse sperm motility. **a** Chromatogram of the analytical C18 RP-HPLC fractionation of the Egyptian snake venom. Most compounds eluted between fractions 8 and 19. Fraction numbers are indicated on top. Dashed line represents the linear ACN gradient. **b** Schematic diagram illustrating the sperm motility parameter (VCL) that was calculated by computer-assisted semen analysis (CASA) system. Positive fractions were those that exceeded +20% variations for VCL (fraction 11). Dashed lines indicate meaningful variations as set arbitrarily for defining potent compounds. **c** Mass characterization of fraction F11 components by MALDI-TOF MS. Five compounds were identified using the linear positive mode

In an earlier report using *W. aegyptia* venom, we illustrated that RP-HPLC remains a method of choice for a well-resolved separation of individual compounds [28]. The venom was therefore separated by analytical C18 RP-HPLC and the venom compounds eluted with a gradient of acetonitrile. The eluate was recuperated into fractions according to the chromatogram illustrated in Fig. 1a. As observed, most compounds eluted between fractions 8 and 19 according to the absorbance measured at 214 nm (peptide bond). Hence all these fractions were first lyophilized, resuspended in 100 μL of dH_2_O, and 10 μL of each fraction was tested on OF1 male mouse sperm motility using the CASA system as described in the *Methods* section. A focus was put on curvilinear velocity of the sperm (VCL) which is the most meaningful parameter to investigate when dealing with sperm motility. While fractions 13 and 14, and to a lesser extent fraction 15, stood out as potent inhibitors of sperm motility, only fraction 11 showed promising evidence for sperm motility activation with an average stimulation of 33.41% (Fig. 1b). The VCL value grew up from 106 ± 27 μm/s (*n* = 68 sperms, mean ± SD) to 141 ± 45 μm/s (*n* = 74 sperms). Variations that occurred at ±20% were arbitrarily considered of interest, while fractions that had lower modulatory functions were disregarded. Hence, fraction 11 was analyzed by MALDI-TOF MS in the linear mode and led to the detection of five ions with *m/z* values ranging between *m/z* 6389.9 and *m/z* 7605.9 (Fig. 1c).

**Fig. 2:**
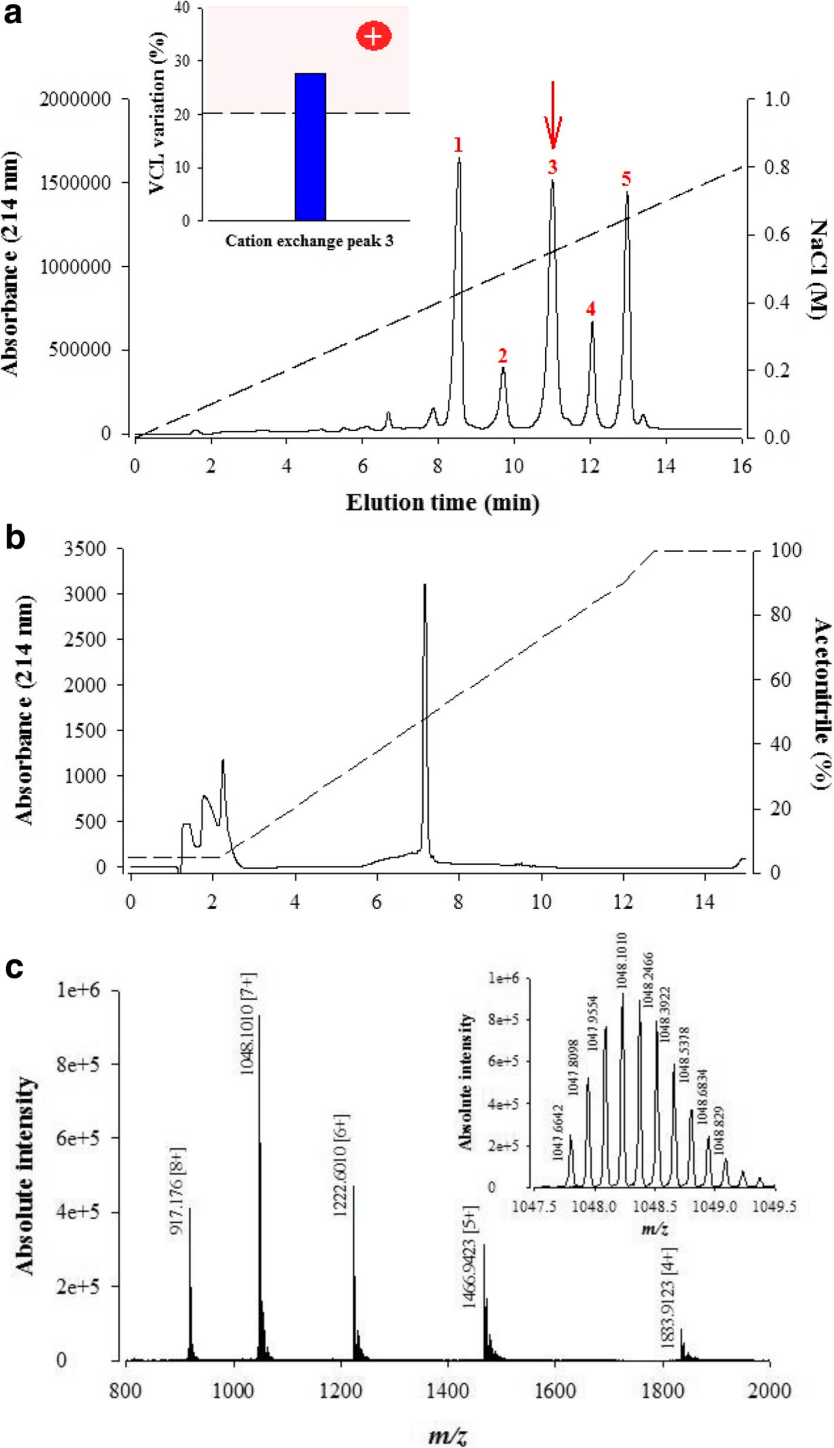
Bioactivity of purified F11 compounds. **a** Cation exchange chromatogram illustrating the purification of five compounds from F11 fraction (from 1 to 5 in red). The dashed line illustrates the NaCl gradient used for elution of the compounds. Minor peaks (two before compound 1 and one after compound 5) were not purified. (**Inset**) Secondary screening of the cation exchange compound 3 (red arrow) on sperm motility parameter VCL. **b** RP-HPLC purification of cation exchange compound 3. Peaks at elution time 2 min are injection artifacts. **c** LC-ESI-QTOF MS of compound 3. Inset illustrates the MS of *m/z* 1048.1010 [7+]

To identify which compound of fraction 11 is responsible for the sperm motility activation, fraction 11 compounds were further individually purified by cation exchange chromatography (Fig. 2a). As illustrated by the chromatogram, five compounds eluted along the NaCl concentration gradient and out of primary fraction 11. Assuming closely related molecular masses for the compounds of fraction 11, which seems to be the case (see Fig. 1c), it appears that compounds 1, 3 and 5 are present in greatest abundance in fraction 11 according to the absorbance values at 214 nm. In contrast, compounds 2 and 4 stand out as the least abundant ones within this fraction.

After desalting, each one of these five compounds were tested on the sperm VCL parameter at a concentration that should closely mimic the initial concentration within the RP-HPLC fraction 11 if one takes into account a recovery of these compounds of about 80% from this fraction following cation exchange. This recovery was assessed using reference compounds (not shown). Compound 3 turned out to be a positive modulator of VCL (inset, Fig. 2a). In this batch of OF1 mouse sperm, the VCL value increased from an average 202 ± 85 mm/s (*n* = 43 sperms) to 257 ± 73 mm/s (*n* = 45 sperms), which represents an average increase of 27.2%. This increase matches the increase in VCL induced by RP-HPLC fraction 11 itself indicating that cation exchange peak N^o^ 3 largely recapitulates the effect of the full fraction 11. As shown by analytical C18 RP-HPLC, the peptide has been purified to homogeneity (Fig. 2b).

LC-ESI-QTOF MS analysis illustrates several charge states of the compound (4+ to 8+) (Fig. 2c) leading to an experimental molecular mass of 7329.71 Da, which is very closely related to one of the components detected in RP-HPLC fraction 11 that had a molecular mass of 7329.8 Da as detected by MALDI-TOF MS (Fig. 1c). This compound was termed actiflagelin in reference to its activation potential of sperm motility, which should be related to flagellum function. Besides compound 3, compound 1 was also active on sperm motility and will be characterized and treated in a separate report (data not shown). This approach does however illustrate that this fraction 11 contained two compounds that both acted positively on sperm motility.

**Fig. 3:**
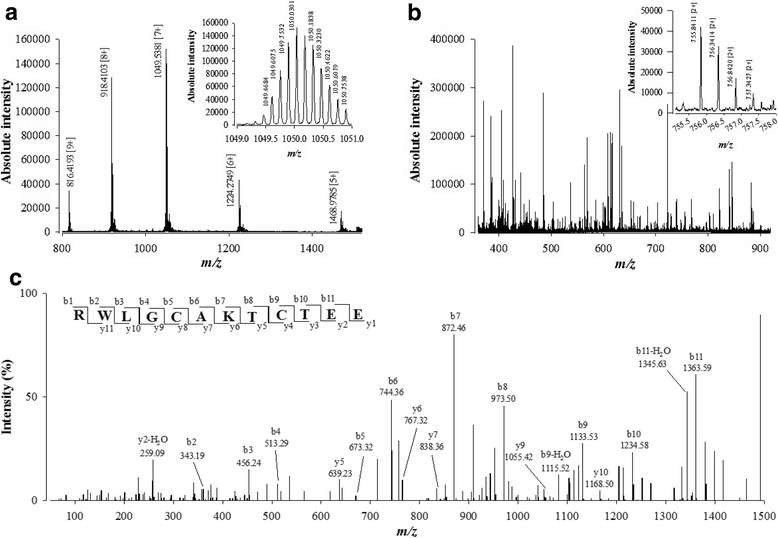
De novo sequencing of compound 3 from fraction F11 by LC-ESI-QTOF MS/MS after reduction, alkylation and protease digestion. **a** LC-ESI-QTOF MS of compound 3 after reduction and alkylation. This allows the calculation of the number of disulfide bridges. **b** LC-ESI-QTOF MS of compound 3 after reduction, alkylation and V8 protease digestion. Inset illustrates the MS of *m/z* 1509.6704 [1+]. **c** LC-ESI-QTOF MS/MS of precursor ion *m/z* 1509.6704. The peptide sequence RWLGCAKTCTEE is derived by de novo peptide sequencing. Peaks corresponding to the y and b ions from this peptide are labeled on the spectrum

The most logical follow-up step was de novo sequencing of actiflagelin. As a first step, actiflagelin was reduced using TCEP for 1 h at 55 °C and alkylated with iodoacetamide for 1 h at room temperature and in the dark. The alterations in molecular mass (from 7329.707 Da to 7899.907 Da), taking into account the reduction of disulfide bridges and the addition of 57.02 upon alkylation, illustrates that actiflagelin should contain ten cysteine residues. In addition, the differences in molecular masses would also suggest that all these cysteine residues engage in five disulfide bridges (Fig. 3a).

Samples of the reduced/alkylated actiflagelin were then digested overnight at 37 °C with either trypsin (*w*/w trypsin/actiflagelin with a ratio of 1/20) or V8 protease (w/w V8 protease/actiflagelin ratio of 1/20). All digested fragments were purified by ZipTipC18 and first analyzed by LC-ESI-QTOF MS. Trypsin generated the following ions of *m/z* 365.6490 [+2], 509.2344 [+2], 469.2189 [+2], 309.1419 [+2], 431.2254 [+2], 367.6878 [+2] and 598.7563 [+2], while V8 protease generated different ions of *m/z* 821.3420 [+3], 755.8411 [+2] and 1082.4535 [+2]. Part of the peptide fragments resulting from V8 protease digestion of actiflagelin are shown on an expanded part of *m/z* ions in Fig. 3b. The MS of ion *m/z* 755.8411 [+2] is also shown (Fig. 3b, inset).

**Fig. 4:**
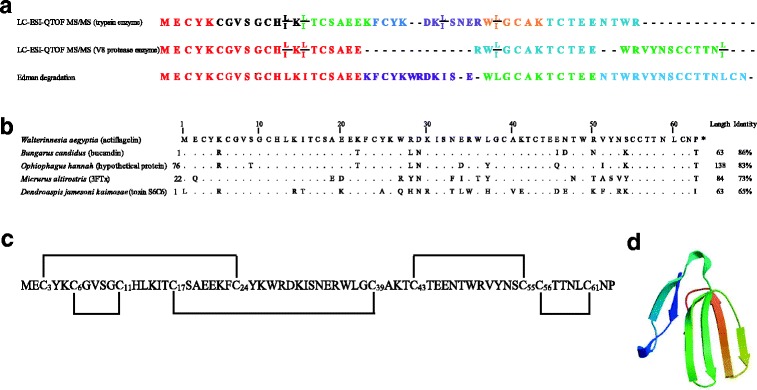
Actiflagelin amino acid sequence, disulfide bridge arrangement and putative structure. **a** Different sequences of actiflagelin that were obtained after MS/MS analyses of the reduced/alkylated/digested peptides and Edman degradation. In MS/MS de novo sequencing Ile and Leu residues cannot be resolved based on the CID activation mode and are therefore labelled (I/L on top of each other). **b** Sequence alignment of actiflagelin with homolog toxins retrieved from the protein BLAST. Hyphen-minus represents identical amino acid residues, and dots indicate the lack of residue at the position. The peptide lengths and percentages of sequence identities are given on the right. **c** Disulfide bridge organization of actiflagelin (in black) proposed by homology with bucandin. **d** SWISS-MODEL (http://swissmodel.expasy.org/) proposed a 3D-structure of actiflagelin

All ions were analyzed by LC/ESI QTOF MS/MS and de novo sequencing software (PEAKS® studio version 5.2 software) used to define the amino acid sequence of each actiflagelin fragment. An example of de novo sequencing by LC-ESI-QTOF MS/MS is provided in Fig. 3c for the *m/z* 755.8411 [+2] ion. The resulting sequence of 12 amino acids was RW(L/I)GCAKTCTEE. A total of three peptides could be sequenced using this method following reduction, alkylation and V8 protease digestion (see colored peptide fragments in Fig. 4a). Peak scores for these peptides were 88, 99 and 91% from N- to C-terminus, respectively. At this stage, Ile or Leu residues can be resolved by LC-ESI-QTOF MS/MS and are thus described as (L/I).

Similarly, seven peptides could be sequenced after trypsin digestion of reduced/alkylated actiflagelin (Fig. 4a). While the use of both proteases allowed for the identification of overlapping sequences, thus permitting the reconstruction of most of the full-length sequences, there remained uncertainties about the central sequence and the extreme C-terminus of actiflagelin. In addition, we could not define whether we had Leu or Ile residues at defined locations within the primary structure since LC-ESI-QTOF MS/MS based on CID activation mode does not discriminate between these two entities. To solve these issues, purified actiflagelin peptide fragments from V8 protease digestion were sequenced by Edman degradation, which yielded additional four peptide sequences, this time covering the central region of actiflagelin and providing most of the C-terminus of this toxin (Fig. 4a). The Edman degradation peptides unambiguously identified Leu residues at positions 13, 37 and 60, while it identified Ile residues at positions 15 and 31. The presence of ten Cys residues (Fig. 4b) is coherent with the conclusion drawn that there should be five disulfide bridges within actiflagelin upon mass shift observed with the full reduction and alkylation of the peptide (Fig. 3a).

The partially reconstructed sequence has a theoretical molecular mass of 7233.22 Da assuming that the five disulfide bridges are connected and that the peptide is not amidated at the C-terminus. Thus, there is still a missing molecular mass gap of 96.48 Da that is compatible with a C-terminal Pro residue at the end of actiflagelin (115.13 Da −18 Da of H_2_O when engaged in peptide bond = 97.13 Da). If, in addition, the peptide is amidated (−1 Da), then Pro would perfectly match the missing residue in the sequence to provide the correct missing mass. This, in turn, would reach a theoretical mass of actiflagelin that matches the observed experimental mass of our sperm motility activator.

Finally, we performed a Blast search using the almost fully reconstructed sequence of actiflagelin, which yielded a match with several animal toxins (Fig. 4b). Sequence identity with these matches ranged between 65% for toxin S6C6 and 86% for bucandin. The sequence of actiflagelin was deposited in the UniProt database (http://www.uniprot.org/help/submissions) with a SPIN ID number of SPIN200010115. The proposed disulfide bridge pattern of actiflagelin is provided in Fig. 4c based on its sequence similarity with bucandin. Finally, a 3D model of actiflagelin built by the SwissModel automatic modeling protein software is shown and illustrates that actiflagelin is a three-finger toxin (Fig. 4d).

## Discussion

Herein, we demonstrate the value of phenotypic screening on an exotic cell type (mouse sperm) that is not predicted to be a normal venom target in toxinology. We followed a two-step purification procedure using RP-HPLC as first step fractionation and cation-exchange for the final purification of the compounds of interest. We based our assay on two simple reasoning schemes: (i) sperm contains diverse and high levels of cell surface receptors and ion channels [13, 29, 30], and (ii) any animal venom is known to contain compounds that efficiently target these two classes of cell surface receptors [31–35]. Hence, it is logic that by taking randomly any kind of animal venom and by screening for compounds that would alter sperm motility, a process governed to a large extent by ion channels and other types of cell surface receptors, we should detect interesting compounds with modulatory function.

By following our purification scheme, we identified one activating primary fraction (11) and two inhibitory ones (fractions 13 and 14) demonstrating the richness in sperm-modulating compounds that should be present in *W. aegyptia* venom. The inhibitory fractions will remain interesting lead fractions for the discovery of at least two additional compounds that possess contraceptive potential. Globally, this observation further assures that animal venoms comprise rich libraries for the discovery of sperm-modulating compounds.

With regard to the inhibitory fraction 11, it was of interest to determine whether the activity was linked to a single compound, several compounds possessing a similar inhibitory activity or to a synergistic effect of several compounds that, if tested apart, would lack efficiency. Upon purification by cation-exchange chromatography, we found out that at least two compounds activate sperm motility within the single RP-HPLC fraction 11, thereby validating the second hypothesis. Time will tell whether the second compound is of the same family of actiflagelin or whether it significantly differs in sequence and cell receptor. All that can be stated at this level is that this second compound should have a molecular mass that resembles the one of actiflagelin according to MALDI TOF MS analysis.

Snake peptides in the range of 6-7 kDa are notoriously active on GPCR [36, 37]. Defining the molecular target of actiflagelin would be beneficial. It would most likely lead to the identification of a GPCR type that could represent a novel lead target for new screening campaign using this time libraries of small chemical compounds that should be easier to synthesize, more cost-effective, and allow oral intake for the control of sperm motility in infertile men. Such a strategy will require: the successful chemical synthesis of actiflagelin; and the identification of actiflagelin receptors by reverse pharmacology, i.e., by using synthetic actiflagelin as a bait in affinity columns. For the time being, actiflagelin will be useful to assess at a later stage its ability to improve the fertilization outcome in vitro. In addition, chemical synthesis of tagged versions of actiflagelin will help examining the cell surface distribution of its receptor and its relevance to sperm motility problems.

## Conclusions

We demonstrate the power of proteomics for de novo identification of venom components that have been identified through a process of phenotypic screening. This method is applicable for any cell type, function or pathology in which cell surface receptors – such as GPCR or ion channels – are suspected to play an important role.

## Abbreviations

ACN: Acetonitrile
ALH: Amplitude of the lateral head
C: Carbamidomethyl
CASA: Computer assisted semen analysis
DDA: Data-dependent acquisition method
dH_2_O: Distilled H_2_O
GPCR: G-protein coupled receptors
LC-ESI-QTOF MS: Liquid chromatography electrospray ionization quadrupole time-of-flight mass spectrometry
LC-ESI-QTOF MS/MS: Liquid chromatography electrospray ionization quadrupole time-of-flight mass spectrometry/mass spectrometry
MALDI-TOF MS: Matrix assisted laser desorption/ionization-time of flight mass spectrometry
RP-HPLC: Reversed-phase high performance liquid chromatography
TFA: Trifluoroacetic acid
VAP: Average path velocity
VCL: Curvilinear velocity
VSL: Straight-line velocity
